# Overcoming cancer therapeutic bottleneck by drug repurposing

**DOI:** 10.1038/s41392-020-00213-8

**Published:** 2020-07-02

**Authors:** Zhe Zhang, Li Zhou, Na Xie, Edouard C. Nice, Tao Zhang, Yongping Cui, Canhua Huang

**Affiliations:** 1 State Key Laboratory of Biotherapy and Cancer Center, West China Hospital, and West China School of Basic Medical Sciences & Forensic Medicine, Sichuan University, and Collaborative Innovation Center for Biotherapy, 610041 Chengdu, China; 2 Department of Biochemistry and Molecular Biology, Monash University, Clayton, VIC Australia; 3 The School of Biological Science and Technology, Chengdu Medical College, 610083 Chengdu, China; 4 Department of Oncology, The Second Affiliated Hospital of Chengdu Medical College, China National Nuclear Corporation 416 Hospital, Chengdu, 610051 Sichuan China; 5 Cancer Institute, Peking University Shenzhen Hospital, Shenzhen Peking University—the Hong Kong University of Science and Technology (PKU-HKUST) Medical Center, and Cancer Institute, Shenzhen Bay Laboratory Shenzhen, 518035 Shenzhen, China; 6 Department of Pathology & Shanxi Key Laboratory of Carcinogenesis and Translational Research on Esophageal Cancer, Shanxi Medical University, Taiyuan, 030001 Shanxi China; 7 School of Basic Medical Sciences, Chengdu University of Traditional Chinese Medicine, Chengdu, 611137 Sichuan China

**Keywords:** Drug development, Drug development

## Abstract

Ever present hurdles for the discovery of new drugs for cancer therapy have necessitated the development of the alternative strategy of drug repurposing, the development of old drugs for new therapeutic purposes. This strategy with a cost-effective way offers a rare opportunity for the treatment of human neoplastic disease, facilitating rapid clinical translation. With an increased understanding of the hallmarks of cancer and the development of various data-driven approaches, drug repurposing further promotes the holistic productivity of drug discovery and reasonably focuses on target-defined antineoplastic compounds. The “treasure trove” of non-oncology drugs should not be ignored since they could target not only known but also hitherto unknown vulnerabilities of cancer. Indeed, different from targeted drugs, these old generic drugs, usually used in a multi-target strategy may bring benefit to patients. In this review, aiming to demonstrate the full potential of drug repurposing, we present various promising repurposed non-oncology drugs for clinical cancer management and classify these candidates into their proposed administration for either mono- or drug combination therapy. We also summarize approaches used for drug repurposing and discuss the main barriers to its uptake.

## Introduction

Cancer is one of the leading causes of mortality worldwide.^1,2^ Opportunities to help reduce the death rate from cancer through the discovery of new drugs are benefiting from the increasing advances in technology and enhanced knowledge of human neoplastic disease.^3,4^ However, translation of these new drugs into clinical practice has been far slower than expected.^5,6^ Drug development requires an average of 13 years research. In addition to design and production, it is necessary to examine the efficacy, toxicity, and pharmacokinetic and pharmacodynamic profiles of the drug in cell- and animal-based studies.^7,8^ Bringing a single new drug from bench to bedside is expensive, with costs of bringing a new chemical entity to market being estimated at ~USD2–3 billion.^9,10^

A key step in drug development is testing the safety and efficacy in human subjects in clinical trials that normally comprise four phases.^11^ Phase I clinical trials test the new drug for the first time in a small group of people (e.g., 20–80) to evaluate safety (e.g., to determine a safe dosage range and identify side effects). Phase II clinical trials study intervention in a larger cohort (several hundred) to determine efficacy and to further evaluate drug safety. In phase III studies efficacy is then studied in large groups of trial participants (from several hundred to several thousand) comparing the new intervention to other standard or experimental interventions (or to non-interventional standard care). Phase III studies also monitors adverse effects and collects further information that will allow the intervention to be used safely. Phase IV studies occur after the drug has been marketed. These studies are designed to monitor the effectiveness of the approved intervention in the general population and to collect information about any adverse effects associated with widespread use over longer periods of time. In general, if the drug is found efficacious in Phase III trials, it receives FDA approval. However, only one of every 5000–10,000 prospective anticancer agents receives FDA approval and only 5% of oncology drugs entering Phase I clinical trials are ultimately approved.^12,13^ Recently, the escalating cost and timeline required for new drug development means that if drug resistance arises, patients with advanced disease may die before alternative treatments become available.^14,15^

**Fig. 1:**
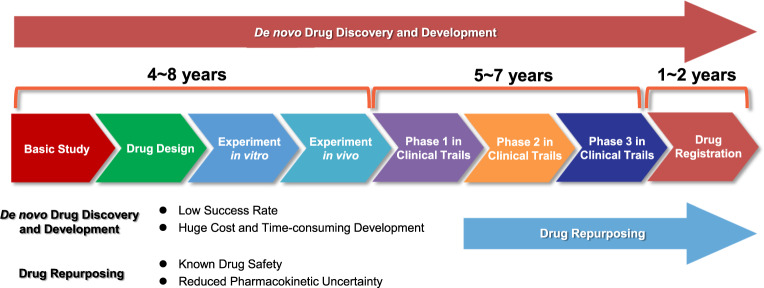
The estimated time and main steps in de novo drug discovery and development and drug repurposing for cancer therapy. De novo drug discovery and development for cancer therapy takes 10–17 years and comprises basic discovery, drug design, in vitro and in vivo experimentation (including identifying safety and efficacy), clinical trials and finally drug registration into the market. In contrast, drug repurposing for cancer therapy takes only 3–9 years as it can bypass several processes that have been completed for the original indication if the anticancer potential of the candidates is confirmed

Drug repurposing (alternatively called “new uses for old drugs”) is a strategy for identifying new uses for approved or investigational drugs that are outside the scope of the original medical indication.^16,17^ Increasingly, researchers and clinicians are considering this strategy to alleviate the dilemma of drug shortage for finding new cancer therapies.^18^ The major advantage of this approach is that the pharmacokinetic, pharmacodynamic, and toxicity profiles of drugs have been already established in the original preclinical and Phase I studies. These drugs could therefore be rapidly progressed into Phase II and Phase III clinical studies and the associated development cost could be significantly reduced.^10,19^ Thus, drug repurposing holds the potential to result in a less risky business plan with lower associated development costs, especially if failures of new drugs during research and development (R&D) are factored in (Fig. 1).^20,21^

In recent years, advanced genomic and proteomic technologies for the assessment of cancer specific biological pathways leading to new drug targets has escalated.^22–24^ This provides excellent opportunities for drug repurposing.^25^ Almost all drugs used in human therapy have the potential to address more than one target.^22,26^ Thus, if the targets of these drugs are highly consistent with cancer, there is a high likelihood that those which share common targets could be therapeutic for other cancer patients.^27,28^ However, drug repurposing historically has been largely opportunistic and serendipitous.^5,29^ Indeed, the several successful examples of drug repurposing to date have not involved a precision therapeutic approach.^30^ Thus metformin, originally an antidiabetic drug, was serendipitously found to be effective in the treatment of various cancers, although the mechanisms for its antineoplastic activity still remain elusive.^31–33^ Undoubtedly the approach based on oncogene targets, due to its precision and flexibility, aids drug repurposing. However, the remarkable heterogeneity of neoplastic diseases poses an obstacle to such strategies.^34–36^ It may be more effective to use an understanding of the “the hallmarks of cancer” to mine “the new tricks of the old drug”, rather than blindly pursue similar targets thus missing out on the “metformin-like” success.

In this review, we will mainly focus on the anticancer activity of existing drugs that were not originally intended for cancer therapy to highlight the relevant signaling pathways and discuss the properties of these agents for the reasonable use of medication based on the hallmarks of cancer. Those targeting sustaining proliferative signaling, resisting cell death, deregulating cellular energetics and avoiding immune destruction may be more effective in monotherapy, while those targeting evading growth suppressors, enabling replicative immortality, inducing angiogenesis, activating invasion and metastasis, genome instability and mutation and tumor-promoting inflammation may be more suitable for drug combination therapy.^37,38^ We also overview the various approaches that contribute to drug repurposing and discuss the major challenges encountered to date in drug repurposing. Overall, we hope that this article may help researchers and clinicians to get a deeper understanding of drug repurposing based on the ten hallmarks of cancer and help translate old drugs into accepted cancer guidelines.

## Non-oncology drugs in drug repurposing

**Fig. 2:**
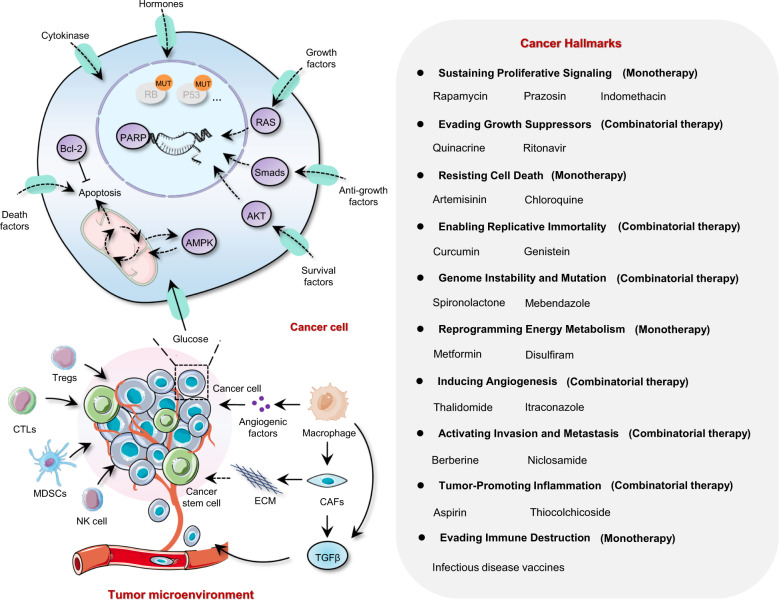
Identification of drug candidates targeting the hallmarks of the cancer cell using drug repurposing enabled by recapitulative signaling networks. The complex signaling interactions contributing to the hallmarks of cancer cells can be orchestrated, rationalizing the complexities of neoplastic disease. Drug candidates interfering with cancer capabilities are shown. CAFs cancer-associated fibroblasts, CTLs cytotoxic T lymphocytes, ECM extracellular matrix, MDSCs myeloid-derived suppressor cells, NK cells natural killer cells, Tregs regulatory T cells

Increasing attention is now being paid to precision medicine, which allows healthcare to be finely tuned to each individual based on molecular profiling instead of the “on drug fits all model” currently used. This has consequentially driven the generation of a biologically compelling lists of genes and proteins based on a systematic analysis of drug discovery for cancer therapy emerging from large-scale multi-omics initiatives.^39–41^ Drug repurposing can target this to find druggable cancer-associated proteins.^28,42,43^ However, a major challenge in cancer drug discovery is to maximize the probability that drugs discovered by either biochemical or phenotypic methods will lead to clinical efficacy and improved disease management.^8,30,44,45^ It should be realized that cell-based models frequently do not accurately represent the true in vivo situation and cancer hallmark-targeting drugs may afford better opportunities for drug repurposing.^46–48^ Key hallmarks of malignancy are clearly not regulated by a single signaling pathway.^37,49^ Hence, mono- or multi-hallmark-targeting drugs have advantages since they may target several supporting pathways pharmacologically, thereby partially avoiding the progression of adaptive resistance.^37,50^ We now summarize non-oncology compounds used for cancer therapy that target the hallmarks of cancer, and distinguish between those agents suitable for monotherapy or as drug combinations (Fig. 2).

### Non-oncology drugs suitable for cancer monotherapy

Many newly identified non-oncology drugs repurposed for cancer therapy act by inhibiting proliferation and inducing cell death as indicated by a large body of data from in vitro and in vivo experiments or clinical trials.^51–53^ In addition, these drugs, which have previously been used for other indications, already have reliable drug safety data and are often inexpensive (especially if they are available as generics).^54^ Some of these drugs are summarized below.

#### Non-oncology drugs which work by inhibiting proliferative signaling

A fundamental trait of cancer cells is their ability to maintain chronic proliferation. Cancer cells can therefore master their own destiny, becoming self-sufficient with respect to growth signaling.^37,55^ Based on an understanding of the underlying cancer biology, various molecular targeting agents, like receptor tyrosine kinase inhibitors, have been developed with excellent success as cancer therapeutics. However, relatively few drugs targeting these pathways have been discovered by drug repurposing.^56,57^ Remarkably, cancer cells may also circumvent pathways that need ligand-mediated receptor induction to sustain proliferative signal, instead relying heavily on three important pro-survival signaling pathways: PI3K/AKT, mTOR, and MAPK/ERK.^58,59^ An ever-increasing number of non-oncology drugs have been repurposed to treat cancer by effective inhibition of these pathways.

##### Rapamycin

Rapamycin allosterically inhibits mTORC1 by binding to the FRB domain of mTOR.^60^ It was approved in 1999 as an immunosuppressant for preventing kidney transplant rejection due to effective suppression of IL-2-mediated T cell proliferation.^61^ In 2002, rapamycin also received clinical approval as an anti-restenosis agent for inhibiting the growth of vascular smooth muscle.^62,63^ However, it was repurposed with the anticancer potential in recent years due to an improved understanding of the role of mTOR and associated signaling networks in cancer.^60,64–66^ Earlier studies reported that the number of leukemic progenitor cells in patients with acute myeloid leukemia was decreased after rapamycin treatment.^67,68^ In addition, rapamycin also showed effective anticancer activation in drug resistant chronic myelogenous leukemia with only mild side effects in most patients.^69,70^ However, more recent studies are highlighting some defects of rapamycin for single-agent therapy. First, the inhibition of mTOR1 induces negative feedback regulation and activation of PI3K-AKT signaling causing survival of cancer cells.^71^ Second, proteins downstream of mTOR such as 4EBP1, a translation repressor protein can be reactivated to drive the proliferation of cancer cells under long-term rapamycin treatment.^72,73^ In addition, rapamycin has limited influence on the activation of mTORC2, which plays an active role in tumorigenesis as an important part of the PI3K–mTORC2–AKT axis.^60,74^ However, some combinatorial strategies of mTOR inhibitors in the clinic have proved beneficial. For instance, the combination of insulin like growth factor 1 receptor (IGF1R) inhibitors with semisynthetic rapamycin analogs inhibits the proliferation of breast and prostate cancers and myelomas.^60,75–77^ Similar encouraging results come from other growth factor receptor antagonists, including the epidermal growth factor receptor (EGFR).^78^ Recently, an AKT inhibitor, perifosine, was used to treat multiple myeloma cells and potentiate the anticancer activity by combination with nanoparticle bound rapamycin.^79^ Furthermore, several clinical trials, in which a RAF pathway inhibitor, sorafenib, or histone deacetylase inhibitor, vorinostat, combined with rapamycin, are underway.^80,81^ It is worth noting that temsirolimus, a rapamycin analog with better solubility and specificity, was approved by the FDA and the European Medicines Agency for the treatment of renal cell carcinoma in 2007.^82^

##### Prazosin

Prazosin was approved for the clinical treatment of hypertension over 40 years ago.^83,84^ The current use of prazosin has been extended to treat multiple clinical indications, such as benign prostatic hyperplasia, Raynaud’s disease and congestive heart failure.^85–87^ Pharmacologically, prazosin is known to be a non-selective inhibitor of α1- adrenergic receptor and can selectively inhibit α2B-adrenergic receptor.^87–89^ Prazosin, showing potential anticancer effects, has been recommended for the treatment of pheochromocytoma.^90,91^ In addition, Desiniotis et al. reported that doxazosin and terazosin, quinazoline-based agents, induced apoptosis by activating the TGF-β signaling pathway rather than adrenergic receptor associated pathways in prostate cancer, suggesting prazosin related drugs, with a well-established and documented clinical record, have potential for cancer therapy by off-target associated drug repurposing.^92^ A recent study indicated that prazosin also induced growth inhibition in a concentration-dependent manner in patient-derived glioblastoma-initiating cells (GICs). Interestingly, there was an off-target mechanism of prazosin in GIC treatment, evidenced by preferential toxicity among other quinazoline-related antagonists of α-adrenergic receptor and activation of the MAPK/ERK signaling pathway followed α-adrenergic receptor blockage. They found that prazosin inhibited GIC proliferation by inhibiting the PKCδ-dependent AKT signaling pathway, but rarely influenced AKT-dependent growth of neural stem cells due to a paucity of PKCδ compared with GICs.^93^ Taken together, these studies imply drug repurposing of prazosin holds promise for clinical cancer treatment.

##### Indomethacin

Indomethacin is a non-steroidal anti-inflammatory drug (NSAID) used for rheumatic disease treatment regimens due to its antinociceptive action.^94,95^ Interestingly, multiple clinical studies have found patients undergoing long term NSAID treatment have a lower risk of developing cancers.^96,97^ In addition, there are an increasing number of reports indicating the antineoplastic effect of indomethacin and indomethacin-based prodrugs against a wide range of cancers by the inhibition of Cox-1/2-dependent angiogenesis.^98,99^ Furthermore, indomethacin has also been shown to reduce the migration and invasion of cancer cells by interfering with calcium-associated signaling and formation of focal complexes.^100^ Several recent studies have uncovered a Cox-independent mechanism of action for the antiproliferative effect of indomethacin, as evidenced by the inhibition of cell proliferation in indomethacin treated colorectal cancer cell lines that do not express Cox-1/2.^101–103^ Recently, Lin et al. suggested that the antiproliferative effect of indomethacin resulted from the inhibition of MAPK-related pathways.^104^ They executed drug repurposing using a library of existing drugs with computational screening. Using binding to the phosphotyrosine binding (PTB) domain of adapter protein Shc (Shc^PTB^) as a readout, indomethacin was confirmed to have a direct interaction with Shc^PTB^. They further showed that indomethacin competes with activated EGFR by binding to Shc^PTB^ without disruption of the ERK-binding site, resulting in a failure of EGFR to recruit Shc to induce aberrant signaling due to the release of ERK. In addition to targeting MAPK pathways, indomethacin also inhibits the proliferation of cancer cells by impairment of PKCζ-p38-DRP1 axis-dependent mitochondrial dynamics or downregulation of Wnt/β-catenin signaling.^105^ Various NSAIDs including indomethacin, have now been proposed as anticancer chemo-preventive drugs.^106–109^ With further understanding of the detailed mechanisms involved in their antineoplastic effect, the clinical application of indomethacin shows much promise.

#### Non-oncology drugs which work by inducing cell death

Apoptosis, which causes cell death once the cell is damaged or faces various physiologic stresses, is a natural barrier for tumorigenesis.^110^ In cancer, deregulated apoptotic signaling, particularly activation of antiapoptotic systems, allows cancer cells to escape this program resulting in uncontrolled proliferation, tumor survival, therapeutic resistance and cancer recurrence.^111^ However, even if tumor cells evolve multiple strategies to antagonize or circumvent apoptosis, other programmed cell death modalities, like lethal-autophagy and ferroptosis, could provide alternative strategies for cancer treatment.^37,55,112^ Increasingly, non-oncology drugs are being repurposed for the treatment of apoptotic resistant cancers by triggering cell death mechanisms.

##### Artemisinin

Artemisinin (ARS) is the active ingredient of artemisia annua L and is used to treat malaria, the world's most prevalent disease affecting over 600 million people each year.^113^ Artemisinin and its derivatives have caught worldwide attention over the past years, and artemisinin-based therapies of malaria are now the established clinical standard.^114–116^ The main mechanism by which artemisinin kills plasmodia is by blocking conversion of hemoglobin to the non-toxic hemozoin in malaria parasites, resulting in accumulation of reactive oxygen species (ROS) from heme-iron.^117–120^

Interestingly, Youyou Tu, who was honored with the Nobel Prize in Physiology or Medicine for first extracting dihydroartemisinin (DHA), found that patients with lupus erythematosus-related nephritis might benefit from DHA due to inhibition of the NF-κB signaling pathway.^121^ In addition, increasing studies reported ARS-related drugs had an unexpected therapeutic effect against viruses (human cytomegalovirus), schistosomiasis and trypanosomiasis.^122–124^ Recent results indicated ARS and its derivatives also had anticancer activity attributed to inducing non-apoptotic programmed cell death.^54,125,126^ For instance, artesunate (ART)-based agents stimulated ROS generation and promoted the lysosome degradation of ferritin instead of autophagic degradation, resulting in system X_c_^-^/GPX4 axis-mediated ferroptosis in cancer cells.^127^ Similarly, Chen et al. found that DHA sensitized cancer cells to ferroptosis by autophagic independent degradation of ferritin, inducing free iron accumulation and subsequently iron homeostasis dysregulation due to binding to the iron-responsive element sequences of iron-regulatory proteins.^128^ In contrast, Du et al. reported DHA-induced cell death associated with autophagic-dependent degradation of ferritin (ferritinophagy) in acute myeloid leukemia cells.^129^ In addition, several targets involved in autophagy of ARS and its derivatives were identified.^54^ Dihydroartemisinin-37 induced autophagic cell death by activating high mobility group box 1 and releasing Beclin 1 from Bcl-2.^130^ Other types of nonapoptotic programmed cell death, including oncosis (ischemic cell death) and anoikis (anchorage-dependent cell death), have also been observed in cancer cells following ARS-based treatment.^131–133^ In recent clinical trials, DHA treatment for advanced cervical carcinoma patients appeared encouraging as clinical symptoms of these patients were relieved and thus survival increased.^134^ In addition, artesunate administered orally in patients with colorectal cancer showed anticancer effects and was generally well tolerated.^135^ 23 metastatic breast cancer patients who were also enrolled to receive ARS-type drugs treatment showed beneficial effects.^136^ Taken together, drug repurposing of ARS and its derivatives, like ART and DHA, offers an opportunity for treating intractable and antiapoptotic cancer by inducing alternative programmed cell death. Further Phase II, Phase III clinical trials will further confirm the suitability of ARSs in clinical oncology.

##### Chloroquine

Chloroquine (CQ) and its derivative hydroxychloroquine (HCQ), which are antimalarial agents in common clinical use, also have therapeutic effect on rheumatoid arthritis and discoid and systemic lupus erythematosus.^137,138^ CQ or HCQ, the only FDA-approved autophagy flux inhibitors, have been used to treated pancreatic and other cancers.^139,140^ Mechanically, CQ or HCQ blocked autophagosome-lysosome fusion through blocking STX17 incorporation by LC3-positive autophagosomes, or impairment of Golgi and endosomal functions, rather than decreasing lysosomal acidity.^141^ Recently, a novel CQ derivative DC661 was reported to have a deacidifying function toward lysosome and had distinct advantages in inhibition of autophagy flux compared with HCQ.^142^ DC661, and other CQ-based drugs, can target and inactivate palmitoyl-protein thioesterase 1 (PPT1), which mediates depalmitoylation to stabilize the v-ATPase subunits located in lysosome. The v-ATPase complex not only maintains the acidity of lysosome for catabolism, but contributes to the localization and subsequent activation of mTOR. In addition, high expression of PPT1 in a variety of cancers was identified using the The Cancer Genome Atlas (TCGA) database, and indicated poor survival.^142^ These findings further confirm the specific targets of CQ-based drugs and broaden the clinical application of CQ-based drugs to patients with tumors exhibiting PPT1-high expression.^142^ CQ-related clinical cancer trials started as early as 1998 when CQ was used for treating glioblastoma multiforme patients showing enhanced patient survival.^143^ Subsequently, CQ or HCQ was used alone or in combination with standard treatments for multiple myeloma, lung cancer, pancreatic cancer, and sarcoma.^144–148^ Drug repurposing of CQ shows considerable promise for autophagy-dependent malignant tumors, including pancreatic cancer and drug resistant cancer mediated by protective autophagy.

#### Non-oncology drugs which work by regulation of cellular metabolism

Reprogramming energy metabolism is ubiquitous in cancer cells, and primarily supports malignancy by sustaining key hallmarks of cancer, including uncontrolled cell proliferation, evading growth suppressors, and resisting cell death.^37,149^ Cancer cells exhibit anomalous energy metabolism, termed aerobic glycolysis, which generates the glycolytic intermediates to facilitate the production of macromolecules and organelles by various biosynthetic pathways. Accordingly, increased aerobic glycolysis significantly enhances glucose uptake and utilization in order to provide essential components required for cell assembly regardless of relatively poor efficiency for ATP generation.^150,151^ In general, reprogramming energy metabolism as a proliferation-inducing phenotype fuels chronic and abnormal cell growth and division.^152,153^ Notably, changes in metabolic patterns may lead to hyper-activation of certain metabolic enzymes that detoxify endogenous xenobiotics derived from abnormal energy metabolism in cancer cells, resulting in the malignant phenotype including resisting cell death and drug resistance.^154,155^ Repurposing existing drugs to target cellular metabolism for cancer treatment is therefore a useful strategy.

##### Metformin

Metformin was approved by the FDA in 1994 for treating obese type 2 diabetes.^156,157^ Generally, patients with diabetes are more prone to several types of cancer, probably due to chronic and increased glycemia contributing in part to tumor development.^158^ A number of studies have shown that diabetes, especially type II diabetes, is closely related to the development of pancreatic, bladder, colorectal cancer and non-Hodgkin’s lymphoma.^159^ Long-term metformin treatment has been shown to substantially lower the risk of breast cancer in women with type 2 diabetes^160^ and to date, more than 100 ongoing clinical trials are investigating the anticancer activity of metformin, using those doses typically used for diabetes, for cancer therapy.^161^ Mechanically, metformin can lower elevated levels of insulin, which activates the PI3K-mTOR signaling pathway, leading to proliferative inhibition of those cancers with insulin receptor expression.^161,162^ Furthermore, metformin has been reported to activate AMPK, a key energy sensor in cellular metabolism, to inhibit cancer cell growth by the negative regulation of mTOR involved in tumor survival.^163^ In contrast, studies have indicated metformin inhibition of mTOR signaling dependent on Ras-related GTPase but independent of AMPK.^164^ Wu et al. further showed that metformin protected tumor suppressor TET2 phosphorylation at serine 99 by activating AMPK, thereby avoiding the destabilization of TET2 and dysregulation of 5-hydroxymethylcytosine. Furthermore, metformin has been shown to induce energetic stress by partially inhibiting oxidative phosphorylation, resulting in repression of some high-energy consumption processes, such as mRNA translation and biomacromolecule synthesis, ultimately contributing to cytostatic or cytotoxic effects.^165^ Recently, Liu et al. reported that metformin mediated tumor cell-intrinsic mitochondrial metabolism in ovarian cancer.^166^ Subsequently, a mitochondrial metabolism-related protein, BTB and CNC homology1 (BACH1), was found to increase expression in triple-negative breast cancer, leading to poor glucose utilization and down-regulated transcription of electron transport chain genes. This study showed that targeting BACH1 sensitized breast cancer to metformin treatment.^167^ Taken together, metformin used in cancer therapy is one of the most successful cases of drug repurposing and several clinical trials, which have advanced to Phase III and Phase IV, are currently investigating the therapeutic potential of metformin in oral, prostate, breast, endometrial, and pancreatic cancers.^168–171^

##### Disulfiram

Disulfiram was originally used in rubber vulcanization, but subsequently has been used for more than 60 years as an alcohol-aversion drug to treat alcohol abuse.^172,173^ Mechanistically, disulfiram inhibits the activation of acetaldehyde dehydrogenase (ALDH), triggering severe nausea and vomiting symptoms contributing to abstinence.^174,175^ In the past years, disulfiram has attracted increasing attention for its anticancer activity both as monotherapy and combinational therapies.^176^ Notably, numerous studies have reported on mechanisms of action of disulfiram that are closely associated with ALDH-related processes of cellular metabolism. For example, Tacconi et al. identified disulfiram-mediated acetaldehyde metabolism as a potential therapeutic target for BRCA1/2-deficient cancer cells, resulting from endogenous acetaldehyde-induced DNA damage.^177^ Furthermore, Choi et al. reported that disulfiram reduced the metabolism of ALDH-positive atypical teratoid/rhabdoid tumor cells. They further showed that the disulfiram-induced change of the nicotinamide adenine dinucleotide ratio of NAD^+^/NADH mediated the function of some NAD^+^-dependent proteins, like SIRT1, which controlled various important processes in cancer cells including apoptosis, cell differentiation and metabolism.^178^ Disulfiram can also mediate formaldehyde metabolism by the blockage of formaldehyde oxidation due to inhibition of ALDH, potentially contributing to the induction of apoptosis in various cancer cells.^179,180^ ALDH has also been reported to play a key role in maintaining the stemness of cancer cells, therefore explaining the anticancer effect of disulfiram in stem-like cancer cells.^181–183^ In addition to ALDH-related processes, disulfiram regulates copper- or zinc-dependent metabolism processes in cancer cells.^84,184^ More and more evidence is indicating that disulfiram-mediated oxidative metabolism partially contributes to its anticancer activity.^185,186^ Notably, clinical trials of disulfiram are ongoing, or have already been completed, for treating patients with melanoma, glioblastoma, breast, prostate, and non-small cell lung cancers (NCT02101008) (NCT01118741) (NCT00312819) (NCT01907165).

#### Non-oncology drugs which work by activation of antitumor immunity

Some cancers, especially virus-induced cancers, can avoid immune surveillance or limit immunological killing by somehow regulating both the innate and adaptive immune systems, which act as an effective barrier to inhibit tumorigenesis and development, to evade eradication.^37,187^ Indeed, increased tumor incidence and cancer development partially attribute to certain deficiencies of immunocytes that include natural killer (NK) cells, CD8^+^ cytotoxic T lymphocytes (CTLs), and CD4^+^ Th1 helper T cells.^37,188,189^ Mechanistically, cancer cells may disable the regulators or effectors of the immune system to evade immune destruction.^190^ An increasing body of evidence suggests that antitumor immunity has a favorable potential to eradicate cancer cells, and is currently revolutionizing cancer care.^191^ However, drugs like immune-checkpoint inhibitors for cancer immunotherapy are not effective in all cases, and would benefit from drug repurposing.

##### Infectious disease vaccines

Vaccines act by stimulating the body’s defense mechanisms against infection to produce the corresponding antibodies.^192^ Recently, mounting evidence has indicated that intratumoral administration of certain infectious disease vaccines exert an anticancer effect by largely eliciting or potentiating tumor immune responses usually manifested as recruitment and activation of immunocytes.^193^ For example, Shekarian et al. reported that rotavirus vaccines activated NF-κΒ and type I interferon pathways in a retinoic acid induced gene 1-mediated manner instead of a Toll-like receptor-dependent manner.^194^ Furthermore, rotavirus vaccines elicited tumor immune responses by enhancing the infiltration of CTLs, NK, and CD4^+^ Th1 helper T cells while the intratumoral administration induced immunogenic cell death, evidenced by ATP release. They also identified the senitization of rotavirus vaccines to overcome anti-CTLA-4 cancer immunotherapy resistance.^194^ In addition, a prophylactic vaccine of yellow fever (live 17D) was shown to exert anticancer activity against cancer cells by the regulation of CTLs and reduction of Tregs.^195^ Interestingly, a previous case–control study reported that anti-influenza vaccines could improve the protective efficacy against cutaneous melanoma, suggesting immune-mediated antitumor activity.^196^ To date, there have been more than 200 clinical trials that utilize vaccines for cancer therapy and an increasing amount of these have advanced into Phase III and Phase IV clinical trials (https://clinicaltrials.gov/). Taken together, attenuated viral vaccines for intratumoral immunotherapy from drug repurposing play a key role in exerting immunostimulatory and oncolytic properties in tumor immunotherapy. Furthermore, it can accelerate the clinical development based on documented safety records. Notably, these approved and marketed agents have shown promising for treating infants and children with cancers.^193^

### Non-oncology drugs suitable for drug combination therapy

In addition to those mentioned above, other compounds may be neglected by researchers for drug repurposing screening due to their low anticancer activity at known tolerated plasma drug doses described in previous indications, hindering their application and development. Fortunately, drugs that may be effective at higher dosage still have a chance to be realized. Indeed, drug combination therapy may be able to utilize such effects and repurpose these drugs, producing a synergistic effect by targeting alternative signaling pathways associated with certain cancer hallmarks, therefore sensitizing cancer cells to other cytotoxic agents. In addition, drug combination therapy may result in reduced doses of the individual agents.^197^ Notably, neoplastic disease has been identified with multifactor and polygenic pathologies, suggesting the requirement to target cancer-related signaling networks using drug combination therapy.^198^ Indeed, drug combinations of two or more compounds is now common practice in the clinic utilizing their diverse mechanisms of action.^199,200^ These drugs will be summarized below.

#### Non-oncology drugs which work by reactivating growth suppressors

Cancer cells can evade mechanisms mediated by the action of tumor suppressor genes.^37,55^ Notably, RB (retinoblastoma-associated) and p53 proteins are two main tumor suppressors, operating in various ways to modulate cells to maintain homeostasis.^201–203^ Defects in function of the RB pathway result in persistent cell proliferation due to dysregulation of gatekeepers of cell-cycle progression.^204^ By contrast, p53 senses intracellular stress, such as energy stress, genotoxic stress, and oxidative stress, preventing further cell-cycle progression until homeostasis returns to normal. In addition, p53 can induce apoptosis when irreparable damage occurs.^205,206^ Accumulating evidence indicates a lack of crucial tumor suppressors (including, but not limited, to RB and p53) stimulating progression to neoplasia.^201,207,208^ More and more non-oncology drugs are being repurposed to target cancer cells evading growth suppressors.

##### Quinacrine

Quinacrine is an antimalarial drug discovered in the 1920s.^209^ It is used to treat giardiasis as an antimicrobial, rheumatoid arthritis, or systemic lupus erythematous as an anti-inflammatory and pneumothorax as a pleural sclerosing agent.^210–212^ In addition, the clinical evaluation of quinacrine for treatment of Creutzfeldt–Jakob disease is in progress.^213–215^ Quinacrine has a convincing drug safety record for long-term clinical use compatible with drug repurposing for cancer treatment.^215^ Indeed, previous studies have indicated quinacrine brings benefits for the treatment of multiple cancers, and its anticancer effects are mediated by p53 activation. Using small molecule screening on renal cell carcinoma cells, quinacrine was shown to induce p53 expression.^216^ In addition, data indicates the cytotoxicity of quinacrine corresponds with increased p53 levels. Subsequent studies suggested that quinacrine-induced p53 expression was possibly mediated by the Facilitates Chromatin Transcription (FACT) protein complex, which is trapped onto the chromatin thereby inducing CK2-mediated phosphorylation of p53.^217,218^ In contrast a recent study reports the inconsistent result that p53 knockdown enhances the quinacrine effect in MCF-7 cells compared with controls.^219^ Regardless, these studies indicate that quinacrine cytotoxicity in cancer cells depends, at least partially, on the p53 status.^220,221^ Reports of quinacrine acting as an anticancer agent in clinical trials are increasing.^222^ For instance, the scientists of Fox Chase Cancer Center currently combined quinacrine with capecitabine for treating colorectal adenocarcinoma in a Phase I, Phase II clinical trial (NCT01844076). Quinacrine has also been combined with erlotinib for the treatment of recurrent or late-stage non-small cell lung cancer in a Phase I clinical trial (NCT01839955). Collectively, quinacrine as an anticancer agent has major potential for cancer therapy and the mechanism may be closely related to activation of p53, a key growth suppressor dysregulated in multiple cancers.^222^

##### Ritonavir

Ritonavir is a protease inhibitor widely used in highly active anti-retroviral therapy (HAART) to treat human immunodeficiency virus (HIV) infection.^223^ HAART, in which protease or integrase inhibitors are combined with reverse transcriptase inhibitors, can substantially slow progression of the AIDS virus rendering it manageable.^224–226^ Interestingly, use of HAART can also result in a reduction in tumor incidence, as evidenced by a Swiss HIV cohort study that reported a decreased incidence of Kaposi sarcoma with HAART treatment.^227,228^ Other research groups subsequently reported similar conclusion on the potential anticancer effect of HAART.^229–231^ In fact, ritonavir as a key component of HAART had already been shown to have anticancer activity in several cancers by inducing apoptosis.^232^ The underlying mechanisms of ritonavir in cancers is controversial but appears to be closely related to key growth suppressors. Gaedicke et al. observed the anticancer activities of ritonavir and accumulation of p53 in ritonavir-treated cancer cells.^233^ A previous study indicated that ritonavir induced cell death of ovarian cancer. Downregulation of CDKs, which inactivate RB through phosphorylation, was observed in the gene profiles of ritonavir treated cells, resulting in RB dephosphorylation and subsequent cell cycle arrest and apoptosis.^234^ A similar outcome of ritonavir-induced G0/G1 arrest was observed in lung adenocarcinoma, associated with downregulation of RB phosphorylation.^235^ Batchu et al. also suggested that ritonavir induced apoptosis in pancreatic cancer, attributing this to inhibition of AKT pathways mediated RB activation and formation of the E2F–1/RB complex.^236^ In a recent phase II study clinical trial, ritonavir combined with lopinavir was used for treating patients with high-grade glioma. However, compared with standard therapeutic regimes, a patient with complete remission showed no improvement in progression-free survival. The authors proposed that the blood brain barrier probably played a key role in the limited efficacy.^237^ Ritonavir as a clinically approved HIV drug has potential for adjuvant therapy of cancer by drug repositioning, although some challenges ahead remain.

#### Non-oncology drugs which work by interfering with replication

In the normal cell lineages, the cell growth and division cycle are controlled by senescence and crisis/apoptosis.^238,239^ In cancer cells, the specialized DNA polymerase, telomerase, which is related to telomere maintenance, is expressed at high levels to counteract this.^240–242^ In addition, TERT, a catalytic subunit of the enzyme telomerase acts as a cofactor to amplify Wnt signaling pathways causing cancer cells to revert to a pre-differentiated stem-like phenotype, eventually exhibiting unlimited replicative potential and substantial stress adaptability.^243–245^ Notably, the Hippo signaling pathways, which control organ size and are dysregulated in cancer, orchestrate Wnt signaling pathways via reciprocal crosstalk with each other by a series of mechanisms.^246–248^ Accordingly, repurposing of drugs which target telomerase to mediate canonical or noncanonical function (influencing Wnt/Hippo signaling pathways), is an actionable and available strategy for improving efficacy of cancer therapeutics.

##### Curcumin

Turmeric, belonging to the ginger family that is widely used in the Asian region as a cooking spice has been used to treat dermatological diseases.^249,250^ Curcumin, the major active ingredient of turmeric, possesses antioxidant, anti-inflammatory, and even anticancer properties.^251,252^ Recently, the influence of curcumin on telomerase has received widespread attention. It has been proposed as a telomerase inhibitor with substantial potential for cancer therapy by drug repurposing. Curcumin has been reported to down-regulate hTERT at the level of transcription to mediate telomerase activity in breast cancer cells.^253–255^ In addition, another study indicated that curcumin inhibited telomerase activity by induction of the dissociation of hTERT from p23, leading to cytoplasmic retention of hTERT and subsequent HSP90-mediated proteasome degradation.^256^ Aravindan et al. showed that curcumin profoundly inhibited telomerase activity by repressing NF-κB binding to the promoter region of the TERT gene following treatment of human neuroblastoma cells with ionizing radiation.^257^ Similarly, a synthetic curcumin analog was shown to affect cancer stemness and telomerase in colorectal cancer. The drug deactivated STAT3 and NF-κB so that the interaction with the hTERT promoter was decreased, resulting in the inhibition of non-canonical functions of telomerase contributing to stemness.^258^ Further studies have confirmed the effects of curcumin on the Wnt/β-catenin and Hippo/YAP signaling pathways, inhibiting stemness or proliferation in multiple cancers.^259–265^ Although limited bioavailability and stability hamper the application of curcumin in clinical cancer therapy,^266–268^ curcumin has been used for treating patients with pancreatic cancer in clinical trials due to its anticancer activity and safety profile.^269,270^ Drug repurposing of curcumin could prove beneficial for clinical translation using highly bioavailable forms of curcumin (e.g., micro- and nano-formulations of curcumin with greatly enhanced absorption) to target telomerase.

##### Genistein

Genistein is an isoflavone found in legumes offering low cost, safe, and dietary accessibility treatment for various pathologies including menopause, osteoporosis, and obesity.^271–273^ Notably, genistein has been used as a phytoestrogen mimic of 17β-estradiol to treat breast cancer.^274^ Subsequently, genistein has been shown to inhibit telomerase activity and related onco-signaling pathways giving potential for treatment of multiple cancers.^275,276^ It has been reported to inhibit transcriptional activation of hTERT by partially attenuating c-Myc in prostate cancer cells.^277^ Genistein inhibits hTERT nuclear translocation posttranslationally, as evidenced by the downregulation of hTERT phosphorylation mediated by AKT in prostate cancer cells.^278^ Genistein has also been reported to inhibit glioblastoma, head and neck and other cancers in a telomerase-dependent manner.^279–281^ In addition, Li et al. discovered genistein-induced telomerase repression due to crosstalk between genetic and epigenetic mechanisms. They indicated that genistein decreased the expression of DNA methyltransferases DNMT1, DNMT3a, and DNMT3b, leading to the hypomethylation of the E2F-1 (repressor) recognition site in hTERT and increased binding of E2F-1 and hTERT. Genistein-induced hTERT reduction has been attributed to increases of the inactive chromatin marker trimethyl-H3K9 and decreases of the active marker dimethyl-H3K4 to the hTERT promoter.^282^ Recently, genistein was reported to inhibit Wnt/β-catenin signaling by regulation of related genes, microRNA, DNA methylation and histone modification, inducing inhibition of proliferation or apoptosis in various cancers.^283–285^ Furthermore, genistein also inhibited stemness, which is closely related to the developmental pathways including Wnt/β-catenin and GDF15, in renal and other cancers.^283,286,287^ Clinical trials of genistein in cancer therapy have been conducted, including in breast cancer (NCT00244933) (NCT00290758), in breast or endometrial cancer (NCT00099008), in bladder cancer (NCT00118040), and in prostate cancer (NCT00005827). However, clinical applications of genistein are still limited due to the poor solubility and bioavailability regardless of its potential anticancer activity by drug repurposing.^288^ Interdisciplinary studies, leading to improved formulation and drug delivery may extend the potential of genistein for clinical anticancer treatments.

#### Non-oncology drugs which work by decreasing angiogenesis

Tumor cells stimulate angiogenesis to generate neovasculature, an important mechanism by which tumors obtain nutrients and evacuate waste products. By contrast, benign neoplasias are relatively dormant, which is attributed to insufficient blood supply.^289,290^ However, when dormant tumor cells are activated by angiogenesis, secreted growth factors induce endothelial cells to bud, leading to a chemotactic response and an angiogenic switch.^291,292^ Antiangiogenesis therapies rely on reducing blood vessel density resulting in tumor starvation.^289,293^ However, antiangiogenic agents are still deficient and usually stimulate cancer to develop stress resistance states.^294^ Accordingly, the identification of more effective drugs with antiangiogenic activity by drug repurposing is important for improving cancer therapy.

##### Thalidomide

Thalidomide is a glutamic acid derivative originally proposed as a sedative in 1957 and was later used as an antiemetic for treating the symptoms of morning sickness in pregnant women. With an improved understanding of the mechanisms of action of thalidomide, it was found to have immunomodulatory, anti-inflammatory, antiangiogenesis and cell proliferation inhibitory properties suitable for treating various diseases.^295–298^ Notably, thalidomide in combination with dexamethasone was approved by the FDA for treating multiple myeloma in 2006.^299^ Further studies have indicated thalidomide has marked anticancer activity in various cancers by mediating angiogenesis.^300^ Nowadays, thalidomide is widely recognized as an antiangiogenic agent that inhibits VEGF, bFGF, tumor necrosis factor-alpha (TNF-α), and various other pro-angiogenic factors.^300^ Thalidomide has been reported to stimulate nuclear export of β-Arrestin1 and trigger aberrant localization of HIF-1α, causing the inhibition of HIF-1-dependent transcription of VEGF-A in breast carcinoma cells.^301^ In addition, thalidomide has been reported to activate sphingomyelinase to generate ceramide, which plays a key role in VEGF-induced angiogenesis, resulting in repression of VEGF receptor expression.^302^ Thalidomide also regulates TNF-α, which could contribute to the decreased squamous cell carcinoma incidence observed in a hamster model.^303^ Furthermore, treatment with thalidomide caused suppression of proliferation, inflammation, and angiogenesis due to inhibition of NF-κB pathways, which were linked to angiogenesis-related cytokine transcription.^304,305^ However, response to thalidomide treatment for solid malignancies, including AIDS-related Kaposi’s sarcoma, gliomas, and renal carcinoma, was modest.^306^ In androgen-dependent prostate cancer, thalidomide showed a significant anticancer effect, with promising performance in a Phase III clinical trial.^307^ Cancer-related clinical trials of thalidomide have been undertaken, regardless of its toxicity issues, while awaiting the development of thalidomide-based analogs with better bioavailable and lower toxicity.

##### Itraconazole

Itraconazole is an FDA-approved traditional antifungal agent with an excellent safety profile.^308^ Unexpectedly, data from preclinical or clinical studies of itraconazole indicated potential anticancer activity in mono- or combination therapies.^309–311^ The anticancer mechanisms of itraconazole probably involve inhibition of resistant protein P-glycoprotein, interference with the tumor microenvironment and mediation of other tumor development-associated signaling pathways.^312,313^ Notably, the antiangiogenesis activity of itraconazole was identified by drug repurposing screening which accelerated its potential use for clinical cancer therapy.^314^ In addition, growing studies are suggesting itraconazole has a multi-target antiangiogenesis effect by regulating various angiogenic signaling pathways.^315^ For example, a study has suggested that itraconazole-induced mTOR inhibition could inhibit angiogenesis via the cholesterol trafficking pathway.^316^ Head et al. further demonstrated that itraconazole directly targeted the mitochondrial protein VDAC1 to regulate AMPK and mTOR, while itraconazole was found to bind to the sterol-sensing domain of NPC1, a lysosomal protein closely associating with cholesterol trafficking, resulting in comprehensive inhibition of cell proliferation and angiogenesis.^317^ Recently, Chen et al. showed that itraconazole significantly inhibited angiogenesis in infantile hemangioma by downregulation of the PDGF/PI3K/Akt/mTOR pathway.^318^ Such studies have led to increased itraconazole-based clinical usage in the past few years. Studies have implied that itraconazole treatment has benefited many patients with ovarian, pancreatic and other cancers.^319–322^ Interestingly, a Phase II clinical study demonstrated that itraconazole combined with standard chemotherapy (pemetrexed) significantly promoted both progression free and overall survival of lung cancer patients, suggesting that the antiangiogenic properties contribute to satisfactory outcomes.^309^ However, some studies have implied certain contraindications for itraconazole, especially interference with other cancer drugs, like rituximab.^323,324^ Despite this, itraconazole is still a promising anticancer agent for clinical application because of existing preclinical or clinical data and drug safety profiles.

#### Non-oncology drugs which work by suppression of invasion and metastasis

Tumor invasion is the mechanism by which tumor cells spread to the surrounding environment, while tumor metastasis is where cancer cells leave the primary tumor and migrate to a new location where they generate new (secondary) tumors. These activities involve regulating existing cellular mechanisms, such as adherens junction signal transduction pathways.^325^ In contrast, defects in these processes can systemically cause tissue abnormalities and disrupt homeostasis, leading to genetic abnormalities or invasion and metastasis of tumors. Accordingly, more and more existing drugs with potent activity against metastatic cancer are being identified by drug repurposing, with the potential of greatly improving the survival of critically ill patients.

##### Berberine

Berberine is an isoquinoline alkaloid present in plants. It is a traditional Chinese medicine and was originally used for the treatment of bacterial diarrhea.^326^ With an increased understanding of berberine, its effect on down-regulation of lipid levels was found to relate to the inhibition of HMG-CoA reductase with benefits for patients with nonalcoholic fatty liver.^327,328^ In addition, berberine was reported to reduce glycemic index by enhancing insulin action or restoring insulin sensitivity, by inhibiting AMPK activation, or upregulating InsR expression, respectively.^329–331^ Recently, numerous studies have indicated that berberine exerts anticancer activity by preventing invasion and metastasis in breast and colorectal cancer and other malignancies. For example, a previous study indicated that berberine modulated ephrin-B2 and inhibited MMP-2 and MMP-9 expression by downregulating TGF-β1, resulting in repression of cell proliferation and metastasis in breast cancer.^332^ A similar outcome from a parallel study showed that berberine inhibited the expression of MMP2 and MMP9 by the regulation of the COX-2/PGE2–JAK2/STAT3 axis.^333^ In addition, berberine inhibited EMT by the inhibition of the RARα/β-mediated PI3K/AKT signaling pathway in melanoma.^334^ In endometrial cancer, berberine was shown to inhibit migration, invasion and metastasis by transcriptionally upregulating miR-101, resulting in inhibition of COX-2/prostaglandin E2 (PGE2) signaling pathways.^335^ Interestingly, berberine can also mediate angiogenesis, whose activation is closely linked to cancer metastasis, by directly reducing VEGF mRNA expression, inhibiting HIF-1α-induced VEGF expression or compromising the PI3K/AKT signaling pathway.^336,337^ Berberine has been used in clinic as an antidiabetic or hypolipidemic agent that has attracted substantial attention.^338–340^ Regardless of poor bioavailable by oral administration, drug repurposing of berberine as a potential anticancer agent is being currently investigated in clinical trials (NCT03281096) (NCT03333265).

##### Niclosamide

Niclosamide is an FDA-approved antihelminthic drug that has been listed as one of the most crucial drugs by the World Health Organization (WHO).^341^ Mechanistically, niclosamide treatment can inhibit glucose uptake and anaerobic metabolism of cells, suggesting a potential effect against cancer cells by targeting tumor metabolism.^342^ Recently, niclosamide has been shown in a number of studies to exert anticancer activity in colorectal, breast and ovarian cancer, and other malignancies.^343–346^ The mechanisms of the niclosamide-induced anticancer effect may be related to multiple signaling pathways, such as Wnt/β-catenin, STAT3, and NF-κB, contributing to cancer invasion and metastasis.^347,348^ In colorectal cancer, a calcium-binding protein S100A4 was found to be upregulated and to promote cancer metastasis.^344,349^ Niclosamide can inhibit liver metastasis of colorectal cancer cells by downregulation of S100A4, causing abrogation of the Wnt/β-catenin signaling pathway.^344,349^ Consistent with this, niclosamide has been shown to reduce lung cancer invasion by inhibition of the S100A4/NF-κB/MMP9 axis.^350^ A similar study reported that niclosamide impaired pulmonary metastases of breast cancer cells by reduction of the STAT3-FAK-Src axis.^351^ In addition, niclosamide can reduce the metastatic potential of lapatinib-resistance breast cancer cell by inhibiting EMT and alleviating stemness phenotype.^352^ Niclosamide in enzalutamide-resistant advanced prostate cancer cells reversed drug resistance, migration and invasion by inhibiting IL6-STAT3-AR signaling pathways.^353^ Data from prostate cancer cells following niclosamide treatment indicated that it inhibited tumor invasion by promoting juxtanuclear lysosome aggregation mediated by lysosome acidification.^354^ An increasing number of studies have shown niclosamide has an anti-metastasis effect in oral squamous cell carcinoma, hepatocellular carcinoma, melanoma, and human thyroid cancer.^355–358^ A Phase II clinical trial of niclosamide, in which the drug was applied to patients with colorectal cancer metastases, has been conducted to assess the drug safety and efficacy (NCT02519582). Clinical trials have also been conducted for the treatment of prostate cancer (NCT02532114) (NCT03123978). However, the poor water solubility and bioavailability of niclosamide has hindered its further clinical development for cancer therapy.^359^ Systemic intravenous administration of niclosamide may improve future applications in the clinic.^360,361^

#### Non-oncology drugs which work by disruption of DNA damage response

Genome instability is one of the major characteristics of malignancy, allowing certain favorable mutant tumor genotypes to survive under stress conditions.^37,362^ Recently, epigenetic regulation, such as DNA, RNA, and histone modification, have also been shown to enable tumors to acquire growth advantages leading to genome instability.^363–365^ Traditional cancer therapies are still focused on chemotherapy and radiotherapy, which induce genotoxic stress by triggering DNA damage. However, these therapies result in severe side effects and can stimulate tumors to develop drug resistance or cancer metastasis even if there is brief tumor dissipation. These worse outcomes result from genome instability, inducing abnormal activation of caretaker genes required for DNA maintenance, which are involved in detection and reparation of DNA damage.^366–368^ Accordingly, a method for enhancing the therapeutic index of traditional cancer therapies could be repurposing of drugs as sensitizers of genotoxic therapy to directly inhibit DNA damage response.

##### Spironolactone

Spironolactone, an aldosterone inhibitor originally used as an effective diuretic for the treatment of high blood pressure and edema, was subsequently shown to benefit patients with heart failure.^369–371^ Increasingly, studies have shown the anticancer properties of spironolactone on prostate and breast cancer.^372,373^ Regulation of DNA damage response appears to be the key mechanism of spironolactone in cancer treatment. For example, spironolactone was identified as an effective nucleotide excision repair inhibitor by drug repurposing, promoting chemosensitivity in cancer cells treated with platinum-based drugs. This is due to spironolactone reversibly inducing the degradation of XPB in a ubiquitin-activating enzyme- and proteasome-dependent manner, impairing both basal and activated mRNA transcription of the transcription/repair factor TFIIH.^374,375^ In agreement with this, a recent study also demonstrated that spironolactone could inhibit SIRT2-mediated transcription-coupled nucleotide excision repair, disturbing cisplatin-induced DNA crosslinks in lung cancer.^376^ In addition, the function of spironolactone on reducing homology directed repair frequencies was confirmed. Goldberg et al. found that spironolactone promoted activity of various drugs targeting DNA damage, such as Poly (ADP-ribose) polymerase (PARP) inhibitor and chemotherapeutic drugs involved in DNA cross-linking, by the impairment of Rad51 foci formation.^377^ They also identified that spironolactone could influence DNA double-strand break repair in cancer stem cells.^378^ Interestingly, Guillotin et al. showed another diuretic drug, triamterene, was active against DNA mismatch repair-deficient tumors. They reported that triamterene increased reactive oxygen species (ROS)-mediated DNA double strand breaks by regulating folate synthesis enzyme and thymidylate synthase.^379^ To date, clinical trials of both spironolactone and triamterene, based on mediating DNA damage response, are rare. However, population-wide data from the Prostate Cancer Data Base Sweden indicated a close relationship between spironolactone treatment and reduced prostate cancer risk.^373^ Taken together, spironolactone and triamterene identified by drug repurposing have shown favorable potential for clinical trials using monotherapy or combinational treatment, mediating DNA damage response for cancer therapy.

##### Mebendazole

Mebendazole, a synthetic benzimidazole, was approved by the FDA for treating intestinal helminthiasis by targeting tubulin polymerization.^380^ In the early 2000s, mebendazole was report to act against non-small cell lung cancers by tubulin depolymerization-induced cell cycle arrest.^381^ Subsequently, tubulin polymerization, the original target of mebendazole, was proposed as its main mechanism of action against glioblastoma and gastric cancer.^382,383^ Importantly, it was shown that mebendazole sensitized cancer cells to radiotherapy by regulating DNA damage response proteins. Markowitz et al. reported that mebendazole inhibited the translocation of Chk2 and Nbs1, mediators of DNA double-strand break repair, to the nucleus, resulting in an increasing effect of radiotherapy.^384^ In addition, mebendazole was shown to have radio-sensitizing activity in breast cancer, and decreased the fraction of stem-like cells via the hedgehog pathway, probably regulating glioma-associated oncogene homolog 1 (GLI1) (the downstream effector of the hedgehog pathway) to respond to DNA damage.^385–387^ In further agreement, a recent study reported that mebendazole reduced GLI1 expression in advanced thyroid cancer.^388^ Mebendazole was also shown to have anticancer activity and enhanced cisplatin-induced DNA damage, by inhibition of DNA double-strand break repair, in head and neck squamous cell carcinoma.^389^ Interestingly, benzimidazole derivatives were reported as potential dual inhibitors for PARP-1 and DHODH, two key proteins involved in DNA replication and repair mechanisms, respectively.^390^ To date, several clinical trials investigating the use of mebendazole for cancer therapy have addressed brain tumors, such as high-grade glioma, medulloblastoma, and astrocytoma (NCT02644291) (NCT01729260), which were mainly treated by radiotherapy or chemotherapy. In addition, a clinical trial using combinational treatment with chemotherapeutic agents (vincristine and carboplatin) and mebendazole was conducted for treating pediatric gliomas in Cohen Children's Medical Center of New York (NCT01837862). A similar clinical trial was run for patients with colorectal cancer (NCT03925662). These studies confirmed the potential effect of mebendazole on inhibiting DNA damage repair-related mechanisms. Two case reports of mebendazole treatment in adrenocortical carcinoma and colon cancer have now been published.^391,392^ These data showed marked remission of cancer metastasis with mebendazole treatment with no obvious adverse effects. The safety, low price, and proven anticancer effect of mebendazole support the feasibility of its clinical application as an adjuvant therapy in multiple cancers.

#### Non-oncology drugs which work by targeting tumor-promoting inflammation

Inflammation is typically associated with the whole process of tumorigenesis, supporting and accelerating the progression of the incipient neoplasm into a malignant tumor by generating various bioactive molecules that can influence the microenvironment around the cancer cells.^393^ In general, growth or survival factors, which maintain an active state of tumor growth by inducing proliferative signaling or reducing cell death are released.^394,395^ In addition, both angiogenic factors and metastasis-related proteins support angiogenesis, invasion and metastasis, resulting from the induction of inflammation.^37,395^ Inflammation can also induce the generation of chemicals, like ROS, which may act as a second messenger to mediate certain signaling pathways in cancer cells, accelerating their genetic evolution, and facilitating their acquisition of the basic hallmarks of cancer.^396^ Regulation of inflammation is therefore an actionable strategy for delaying tumor development and improving cancer therapy efficacy. Many existing drugs identified by drug repurposing exert their anticancer properties by mediating inflammation processes.

##### Aspirin

Acetylsalicylic acid, commonly known as aspirin, is a non-steroidal anti-inflammatory drug (NSAID) approved by the FDA for the treatment of pain and fever and subsequently in the treatment of stroke and cardiovascular disease.^397–399^ Aspirin has also attracted attention for its potential use in cancer therapy. The first suggestion that aspirin may have potential anticancer effects was reported almost 50 years ago, as evidenced by the reduction of lung metastases in aspirin-treated tumor-bearing mice.^400^ Multiple studies have investigated the anticancer effect of aspirin in various cancers, and explained the properties by multiple molecular mechanisms. For example, studies have indicated that aspirin has inhibitory effects on COX-1/2, which are closely associated with cancer-related inflammation by stimulating the synthesis of various prostaglandins, such as PGE2.^401,402^ Subsequently, aspirin was demonstrated to kill melanoma, prostate, ovarian and other cancers by inhibition of PGE2 expression.^403–406^ Aspirin was also reported to mediate tumor-promoting inflammation in a COX independent manner, mainly by regulating the NF-κB signaling pathway, which was initially shown as a key event relating to chronic inflammation and increased cancer risk.^407^ Zhang et al. showed that some aspirin-based drugs exerted anticancer activity in colon and pancreatic cancer cells by inducing ANXA1, which could prevent NF-κB binding to DNA leading to increased apoptosis and decreased proliferation.^408^ Furthermore, Huo et al. recently found that aspirin prevented NF-κB activation and CDX2 expression stimulated by acid and bile salts in esophageal squamous cells from patients with Barrett's esophagus.^409^ Aspirin treatment was also reported to enhance COMMD1 acetylation, regulating the ubiquitylation and nucleolar translocation of the RelA NF-κB subunit.^410^ Aspirin can also mediate the production of inflammatory cytokines, such as MCP-1 adipokine and interleukin (IL)-6.^411,412^ Interestingly, aspirin-triggered resolvins and lipoxins, as the specialized pro-resolving mediators, induce tumor cell debris degradation by macrophage phagocytosis and antagonize macrophage-secreted pro-inflammatory cytokines.^413^ Taken together, stimulating preclinical studies supports the notion that drug repurposing of aspirin as an anticancer agent for cancer prevention or treatment has potential. However, aspirin intake must be carefully controlled as it can cause intracerebral and gastrointestinal bleeding, a barrier to the widespread use of aspirin.^414^ Recently, Stegeman et al. demonstrated that people between age 40 and 85 would benefit from aspirin treatment designed to prevent a primary cancer.^415^ In addition, a previous study indicated that a protective effect against gastrointestinal, esophageal, pancreatic, brain, and lung cancer was associated with a low dose of aspirin (75 mg/day).^416^ Subsequently, several studies suggested a favorable effect of aspirin on colorectal cancer.^417,418^ Notably, Li et al. indicated patients with colorectal cancer, especially with positive PTGS2 (COX-2) expression and PIK3CA mutation, would benefit from post-diagnosis aspirin therapy, evidenced by improved overall survival.^419^ Clinical trials on the use of aspirin in cancer therapy are becoming increasingly common worldwide, suggesting uptake of the routine clinical application of aspirin for cancer therapy.

##### Thiocolchicoside

Thiocolchicoside is a semisynthetic colchicoside derived from Gloriosa superba (Liliaceae) and is clinically approved for treating rheumatologic and orthopedic disorders due to an analgesic and anti-inflammatory effect.^420^ Interestingly, several studies have recently mentioned the anticancer properties of thiocolchicoside. For example, Reuter et al. reported that thiocolchicoside exerted significant anticancer activity in multiple cancers, including leukemia, myeloma, and squamous cell carcinoma. Their results indicated that thiocolchicoside reduced the activation of NF-κB and COX-2 by inducing ubiquitination degradation of IκBα, a key inhibitor of the NF-κB signaling pathway mediating IKK status and p65 nuclear translocation.^421^ Subsequently, they further identified the effect of thiocolchicoside on inhibition of cancer-induced bone metastasis with the ability of suppressing receptor activator of NF-κB ligand and the NF-κB signaling pathway.^422^ Notably, several pharmaceutical companies, provide thiocolchicoside (e.g., Muscoril and Myoril) as a myorelaxant and promote its application as an anti-inflammatory and analgesic drug.^423^ In addition, some clinical trials confirmed the drug safety of thiocolchicoside in the treatment of acute lower back pain.^424,425^ Thiocolchicoside, now a half-century old drug, therefore has substantial potential for cancer therapy by drug repurposing regardless of the few clinical trials to date investigating its anticancer activity. Interestingly, clinical trials of colchicine, which is closely related to thiocolchicoside, have been conducted in Kaohsiung Medical University Hospital to investigate its anticancer effect on invasion and metastasis in patients with hepatocellular carcinoma (NCT01935700) (NCT04264260).

**Fig. 3:**
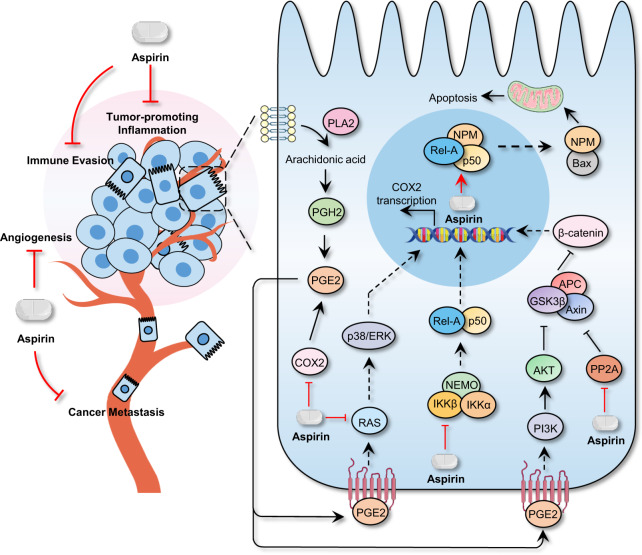
Signaling pathways mediated by aspirin. Multiple signaling pathways regulated by aspirin is shown, acting on diverse hallmarks of cancer including tumor-promoting inflammation, deregulating energy metabolism, angiogenesis, cancer metastasis and immune evasion, are shown

**Fig. 4:**
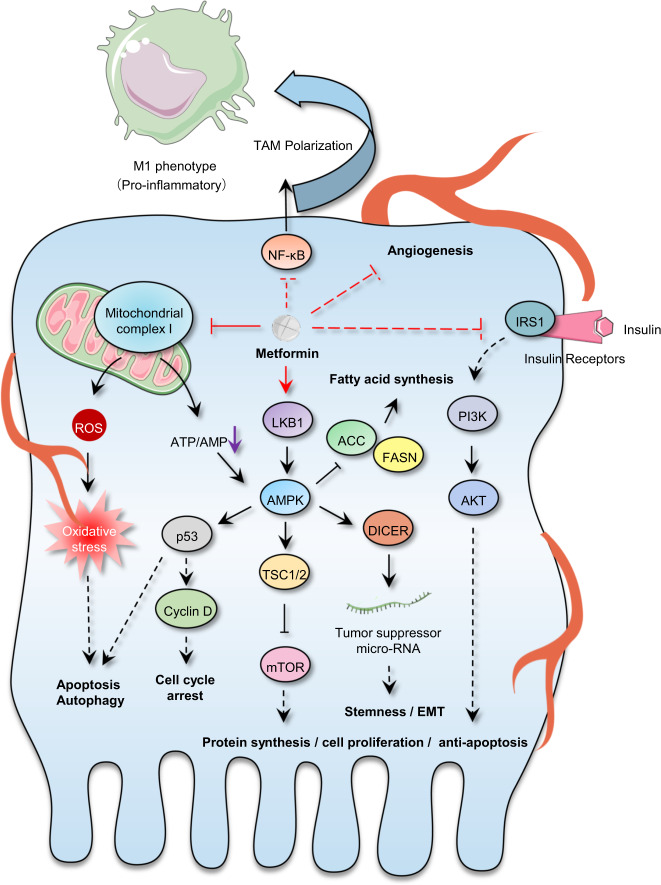
Signaling pathways mediated by metformin. Direct or indirect protein targeting by Metformin is shown. These influences diverse hallmarks of cancer including regulating cell proliferation, self-renewal, cancer metastasis, angiogenesis and energy metabolism

Undeniably, specially targeted drugs with powerful anticancer activity and relatively few off-target effects are responsible for improved patient survival in the clinic.^14^ However, multiple lines of evidence suggest that most of these molecular agents show a transitory tumor therapeutic effect ultimately leading to relapse, because several alternative parallel signaling pathways supporting malignancy can be activated attributing to adaptation of therapy pressure.^37,238,426,427^ In this section, we summarize the potential existing drugs, that were investigated for cancer therapy in ongoing or forthcoming clinical trials, based on the hallmarks of cancer.^37,238,426,427^ Given the shortcoming of targeted drugs that stimulate adaptive resistance, discovery of traditional non-oncology compounds that exert comprehensive inhibitory effect on more than one characteristic hallmark is ideal for developing durable strategies for cancer therapy by drug repurposing. This strategy has already shown efficacy. For instance, aspirin can directly regulate different target proteins, such as COXs, PP2A, RAS, IKKβ, and histone, acting on multiple hallmarks of cancer including tumor-promoting inflammation, deregulating energy metabolism, angiogenesis, cancer metastasis, and immune evasion (Fig. 3).^428^ Metformin has been identified to have potential for mediating various pathways, including insulin/IGF1, NF-κB, AMPK/mTOR/PI3K, Ras/Raf/Erk, Wnt, Notch, and TGF-β signaling, therefore regulating cell proliferation, self-renewal, cancer metastasis, angiogenesis, and energy metabolism (Fig. 4).^429^ Similar outcomes have been obtained with disulfiram, itraconazole, and artemisinin.^54,312,428,430^

**Fig. 5:**
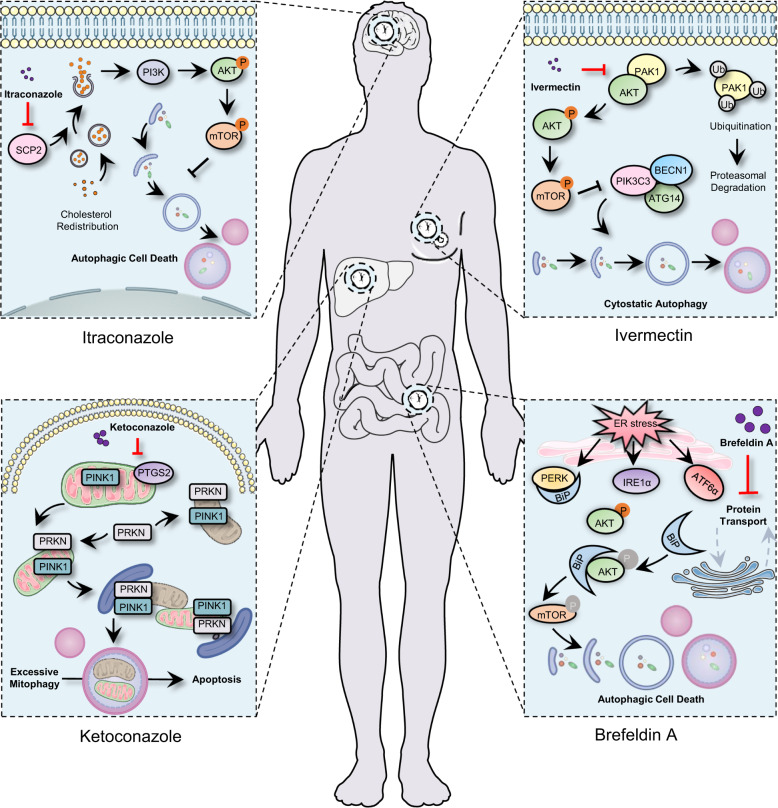
Partially repurposed drugs emanating from our laboratory. In addition to itraconazole, ivermectin, ketoconazole and brefeldin A which target brain, breast, liver and colon cancer, respectively, antifungal agent ciclopirox olamine, anthelminthic drug pyrvinium, and natural products quercetin, 3′-epi-12β-hydroxyfroside and toxicarioside O have also been studied

Indeed, previous studies in our group have repurposed a panel of non-oncology drugs for cancer treatment, including antifungal agents (ciclopirox olamine, ketoconazole and itraconazole), antiparasitic drug (ivermectin), anthelminthic drug (pyrvinium), antibiotic agent (brefeldin A), and natural products (quercetin, 3′-epi-12β-hydroxyfroside and toxicarioside O), showing potential promises for drug repurposing in future cancer therapy (Fig. 5). For example, brefeldin A, a macrolide antibiotic, showed significant autophagic cell death both in vitro and in vivo in colorectal cancer. Mechanistically, brefeldin A provoked endoplasmic reticulum stress-mediated upregulation of binding immunoglobulin protein (Bip), therefore promoting to increased Bip/Akt interaction resulting in decreased Akt phosphorylation, which usually activates autophagy.^431^ Another study found that ketoconazole, a traditional antifungal drug, induced PINK1/Parkin-mediated mitophagy by downregulating COX-2, which resulted in the acceleration of apoptosis, thereby inhibiting the growth of HCC.^432^ Ivermectin, a broad-spectrum antiparasitic drug swept the 2015 Nobel prize for physiology or medicine, displayed anticancer activity against breast cancer cells by inducing PAK1/Akt regulated cytostatic autophagy.^433^ Interestingly, recent studies demonstrate that ivermectin also inhibits the replication of SARS-CoV-2 in vitro, although the paper by Caly et al.^434,435^ has elicited two letters to the editor to question the clinic use for human therapy. In summary, our previous findings, together with others, have laid a solid basis for repurposed non-oncology drugs for cancer treatment. We believe this strategy will achieve great success in cancer drug development.

## Technological approaches to drug repurposing for cancer therapy

**Fig. 6:**
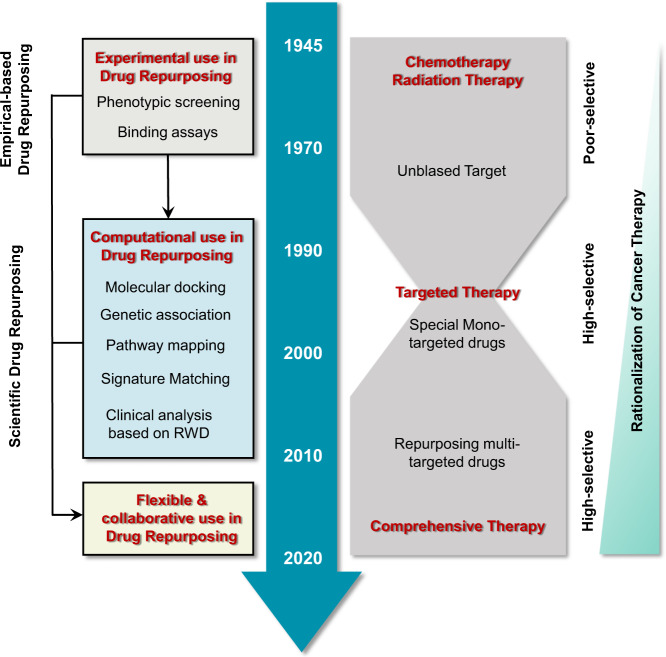
The evolution of screening and therapeutic strategies in drug repurposing for cancer. With the development of biochemical techniques and bioinformatics, treatments based on targeted therapy mainly focus on computational approaches of drug repurposing. Currently, drug repurposing typically depends on a flexible and collaborative use of both experimental and computational approaches to screen multi-targeted agents

Before an old drug can be selected for evaluation as an anticarcinogen with effectiveness and therapeutic potential, a mechanistic assessment of the drug effect in preclinical models is critical and requires systematic approaches.^109,436^ These approaches can be broadly divided into computational and experimental approaches, which employ existing data or biochemical experiments respectively to analyze the possibility that the “old drugs can treat tumors with new tricks”.^437,438^ In fact, success in drug repurposing typically depends on a flexible and collaborative use of both approaches (Fig. 6).^439,440^ The most commonly used computational and experimental approaches and examples of drug repurposing cases are detailed below.

### Computational approaches based on molecular theory (MOT)

Understanding a drug at the molecular level, and matching it with clinical symptoms of neoplasms different from those for which it was originally approved or developed, is key for computational approaches based on MOT leading to drug repurposing.^441–443^ Such approaches involve comprehensive analysis of experimental data, such as chemical or protein structure, gene or protein expression, and various omics data, which assist researchers to put forward a repurposing hypothesis.^444–447^

#### Molecular structure analysis

Using high-throughput computational structural analysis is an efficient way to predict binding site complementarity between the ligand and the target.^439,448^ Docking studies are the main algorithmic approach for predicting the orientation of a ligand into a cavity of the target protein.^449,450^ High-quality structural data of known receptor targets involved in cancer can accurately predict specific drug targets using drug libraries.^451,452^ As an exemplar, bromovinyldeoxyuridine (BVDU) is a thymidine analog used for the treatment of herpes zoster infection, which interacts with viral thymidine kinase using several specific noncovalent binding sites.^453,454^ Heinrich et al. found that heat shock protein HSP27 was a potential target of BVDU, based on a predicted binding site to a viral thymidine kinase and identified some kinase inhibitors targeting HSP27 by in silico docking of a targeted library.^455,456^ Following up on these findings, Salentin et al. used the Protein Data Bank, which contains structural data for more than one thousand different drug targets, to explore any drug–target pairs, which have similar interactions with BVDU and thymidine kinase.^457,458^ The antimalarial drug amodiaquine was found to inhibit HSP27 chaperone function and reverse tumor drug resistance in multiple myeloma cell lines. Binding site comparisons are also a popular approach to predict similar pharmacological drugs.^453^ Lim et al. predicted the heart failure drug levosimendan as an inhibitor of RIOK1 and other kinases by using ligand binding site comparison and protein-ligand docking. They went on to show that levosimendan had anticancer effects by directly inhibiting RIOK1 and RNA processing enzymes.^459^

#### Signature or pathway matching

Various omics studies of cancer not only deepen our understanding of tumor hallmarks at the molecular level, but also provide big data for supporting drug repurposing by applying advanced bioinformatics.^460,461^ A detailed framework constructed by computer processing can integrate the mechanisms of drug action, phenotypes and molecular biological characteristics of cancer to identify novel drug–disease relationships and indicate potential drug repurposing possibilities.^439,444^ The novel associations between genes and cancer found by Genome-Wide Association Studies and Phenome-Wide Association Studies have expanded our knowledge of new targets for existing drugs.^462–464^ Drugs targeting cancer are inferred by overlapping cancer-associated genes and drug-targeted genes from DrugBank, a freely accessible database comprising over 13,000 drugs and related pathways or targets (https://www.drugbank.ca/).^465–467^ Medication Indication Resource (MEDI), another freely-available, computable MEDI can be used to confirm the plausibility of inferred drug indications, which have clinical potential.^468,469^ Following this approach, alosetron was discovered to have genetic correlation with bladder cancer although originally used to treat irritable bowel syndrome.^470,471^ Bezafibrate, a lipid regulating agent, was also suggested to have anticancer properties for melanoma.^472^ In a similar approach, Xu et al. utilized The Cancer Genome Atlas (TCGA) transcriptomes to construct non-tissue-specific core signatures, which were used to identify drugs whose perturbation signatures mimicked a single gene mutation.^473^ Combined with the use of the drug treatment profiles and an in silico screen, results indicated that several psychiatric drugs (such as trifluoperazine) and antihypertensive calcium channel blockers (such as perhexiline) might have anticancer potential by inhibition of the TGF-β pathway or modulation of AKT and AMPK phosphorylation, respectively.^473,474^

### Computational approaches based on Real World Data (RWD)

RWD, as its name suggests, is mainly composed of the real electronic health records (EHRs) of patients characterized by large and complex datasets.^475–477^ EHRs are the systematized collection of electronically-stored patient and population health information in a digital format^478^ and contain large amounts of data on patient outcomes.^479,480^ These are divided into diagnostic and pathophysiological data, including experimental data, drug prescribing data, disease phenotype, and imaging data.^475,481^ Both structured and unstructured data from patients have considerable value as a source for drug repurposing by identifying consistent signals.^482^ Moreover, the plentiful amount of EHR data allows for significant statistical significance.^483,484^ Retrospective clinical analysis is the most commonly used computational approach based on RWD and the data are mainly obtained from EHRs.^485,486^ A classic case of repurposing a noncancer drug for cancer treatment from retrospective clinical analysis is that of metformin which can decrease cancer mortality in a dose dependent manner.^487,488^ Xu et al. designed a clinical cohort from 15 years of EHRs from Vanderbilt University Medical Center and Mayo Clinic. In the independent populations, data from over 100,000 patients with a cancer diagnosis were used in the study and were validated with beneficial effects of metformin for cancer survival, including breast, colorectal, lung, and prostate cancers.^489^ Valproate, previously approved for use in epilepsy, also has been successfully repurposed arising from retrospective clinical analyses for the treatment of acute myeloid leukemia and glioblastoma.^490–492^

### Experimental approaches based on intermolecular interactions

Proteomic techniques using affinity chromatography combined with mass spectrometry, showing protein interactions based on intermolecular force have been widely used for target validation, facilitating drug repurposing due to an experimentally based pharmacological analysis.^493,494^ In this approach, cells or animals are treated with drugs followed by deep proteome analysis to differentially quantify proteins changes that occurred.^495,496^ With the development of labeling (including metabolic labeling and chemical labeling) or label-free approaches, proteins targeted by the drug can be detected in an unbiased fashion.^497,498^ Gefitinib, an epidermal growth factor receptor kinase inhibitor was identified to have more than 20 targets by incubating cancer cells with a covalently modified gefitinib followed by mass spectrometry.^499^ The Cellular Thermo Stability Assay, based on the principle of altered protein thermal stabilization/destabilization in response to ligand binding enables the identification of drug targets by estimating the interaction of target proteins and drug-like ligands.^500,501^ By combining this with thermal proteome profiling, which can be applied in living cells without requiring compound labeling, cellular targets can be confirmed in an unbiased manner.^502–504^

Approaches based on chemical genetics are also dependent on intermolecular forces, and can help explain the relationship between binding and drug efficacy.^505,506^ An example of the success of this technique comes from Karaman et al. They evaluated several kinase inhibitors against pathologically significant human protein kinases using a competition binding assay in vitro. Interaction maps for 38 kinase inhibitors across a panel of 317 kinases representing >50% of the predicted human protein kinome generated a heat map of 3175 binding interactions. Surprisingly, some drugs including but not limited to sorafenib showed higher affinity to other targets than the published known targets. In addition, different types of targets of existing drugs, such as the anthelmintic drug niclosamide to treat Zika virus infection, are becoming increasingly recognized due to the development of chemical genetics suggesting repurposing opportunities for cancer.^507^

### Experimental approaches based on phenotypic screening

Phenotypic screening is a direct way of drug repurposing by analyzing the relative effects in a designed model even if previous studies have not identified the candidate drugs targets.^8,44^ Typically, a series of cell based in vitro assays in 96- or 384-well format are used in phenotypic screening.^508^ For example, Iljin et al. identified disulfiram used for alcohol abuse as an antineoplastic agent by conducting high-throughput cell-based screening, using proliferation as the phenotypic criteria, with a library of 4910 druggable small molecules against four prostate cancer and two nonmalignant prostate epithelial cell lines. Subsequently, they validated the anticancer effect of disulfiram using genome wide-gene expression studies.^509^ Recently, Corsello et al. reported their studies on drug repurposing from a viability evaluation of 578 human cancer cell lines of diverse tumor types treated with more than 4500 drugs including hundreds of non-oncology compounds. They utilized a novel method named “PRISM” (profiling relative inhibition simultaneously in mixtures), which tracks the proliferation of mixtures of cancer cell lines during treatment with the candidate drug using unique molecular barcodes. If a particular drug has efficacy, proliferation of cell lines will decrease, resulting in depletion of a specific molecular barcodes. Using a bead-based assay, the relative abundance of each barcode was determined and anticancer activity profiles generated. 49 non-oncology drugs had an unexpectedly high rate of anticancer activity.^510,511^ Whole animal screening assays can also be utilized in drug repurposing. Whole organism phenotypic assays not only identified candidate drugs effective against cancer, but additionally yielded pharmacokinetic and organ-toxicity results compared with cell-based screening.^512–515^ Ridges et al. used genetically engineered T-cell reporting zebrafish as a platform to evaluate over 26,000 small molecules for efficacy against leukemia and found that 1H-indole-3-carbaldehyde 8-quinolinylhydrazone, termed lenaldekar, showed significant activity against various hematologic malignancies, including T-acute lymphoblastic leukemia and chronic myelogenous leukemia.^513,514^

## Challenges in the development of drug repurposing

Drug repurposing, aiming to repurpose old compounds for new indications, is already in widespread use in cancer therapy. However, few repurposed drugs are officially included in the published cancer clinical practice guidelines.^16,516,517^ In spite of advantages such as demonstrated anticancer pharmacokinetic properties, and acceptable safety and tolerability in humans, there is (as in all drug development) still a possibility of failure in later stages of clinical trials because of competition from successful new drug development.^16,517^ Other barriers include legal and regulatory issues such as patent-related considerations and inequitable prescription charges.^16,516,517^ Hopefully such barriers will prove surmountable.

### Patent considerations

A repurposed product for cancer therapy could bring substantial profit to the patentee. However, the intellectual property status of these candidates is usually ambiguous and unpatentable as the scientific literature or clinical practice is prior art. Accordingly, only non-profit organizations are interested in developing such drugs as returns on investment could be low. In the Repurposing Drugs in Oncology (ReDo) project that includes 72 drugs involved in 190 registered clinical trials, <5% are sponsored by the pharmaceutical companies with the non-profit organizations represented by universities or hospitals supporting the remainder.^517,518^ For drugs that are off-patent, a new method-of-use patent can be obtained for a new repurposed use of an old generic drug. However, the patent application process is strict, requiring detailed data showing proof of credible treatment for the concerned indication, probably using unique formulations and dosage forms. Furthermore, if the generic manufacturer challenges those patents lengthy and costly delays may ensue. Consequently, patent consideration-induced market exclusivity has been identified as a key hurdle in drug repurposing.^16^ Recently, the Off‑Patent Drugs Bill 2015–16, was put before the UK Parliament to address this. It was supported by a number of medical charities but failed to pass into legislation.^16^

### Inequitable prescription

Theoretically, drugs prescribed by physicians should take into account scientific evidence based on clinical trials, generic or repurposed drugs should be prescribed where appropriate. However, the pharmaceutical industry can influence this by spending large amounts of money on drug promotion, marketing to physicians and advertising to consumers. Thalidomide, discussed above, is an excellent example of problems that can occur. Originally prescribed as a sedative or an antiemetic with low-cost and safe properties, it was recently repurposed to treat multiple myeloma. In two randomized clinical trials, the therapeutic regimen melphalan–prednisone–lenalidomide was compared with that of melphalan–prednisone–thalidomide, and showed no survival advantage.^519,520^ However, lenalidomide rather than thalidomide was approved as the standard therapy even if its approximate cost is 43-fold times higher than thalidomide. Obviously, prescribing cheaper drugs, with equivalent activity to the expensive ones, benefit patients rather than biopharma.

## Conclusions and future pespectives

Cancer is still the second leading cause of death globally (around 9.6 million deaths in 2018) in spite of extensive studies to find new treatment regimens and more effective drugs.^1^ However, the cost of cancer treatment is increasing, largely due to the expense of taking drugs through clinical trials (typically around $2.6 billion according to a recent study) where there is only a low rate of success (around 12%).^521^ There therefore exists an urgent need to develop effective, safe, cheaper, and readily available anticancer agents. Theoretically, drug repurposing could break the current drug shortage bottleneck. Importantly, our understanding of cancer biology and the associated hallmarks of cancer is increasing.^37,238,426,427^ This, coupled with repurposing studies that apply systematic screening of the entire pharmacopoeia coupled with advanced bioinformatics, should identify new drugs and their targets. In addition, the use of non-oncology drugs, that account for the bulk of our medications, has the potential to further accelerate the progress of drug repurposing. In this review we have overviewed the most commonly used approaches for drug repurposing, addressing both phenotypic-based and target-based strategies. Based on widespread recognition of these concepts and related studies of drug repurposing over the last decade, we have summarized and evaluated old non-oncology drugs as potential candidates for drug repurposing (Table 1). We have discussed how some of these drugs effectively regulate at least one hallmark of cancer, often induced by multiple parallel signaling pathways, while others exert comprehensive anticancer effects by regulating multiple targets mediated by various alternative signaling routes. Their potential for mono- or drug combination therapy is discussed with respect to the key hallmarks of cancer.

**Table 1:** A total panel of repurposed drugs mentioned in this review

Cancer hallmarks	Repurposed drug	Original application	Reported targets/pathways	Clinical trials of cancer
Sustaining proliferative signaling	Rapamycin	Immunosuppressant, anti-restenosis agent	mTOR and associated signaling networks	Rectum, breast, prostate cancer etc.
Sustaining proliferative signaling	Prazosin	Hypertension	PKCδ-dependent AKT signaling pathway	Adrenal incidentalomas
Sustaining proliferative signaling	Indomethacin	Rheumatic disease	Shc-ERK axis, PKCζ-p38-DRP1 axis, Wnt/β-catenin	Colorectal, esophageal, ovarian cancer etc.
Evading growth suppressors	Quinacrine	Malaria, giardiasis, rheumatoid arthritis	p53, FACT-CK2-p53 axis	Prostatic, non-small cell lung cancer etc.
Evading growth suppressors	Ritonavir	Human immunodeficiency virus	p53, CDKs-RB axis, AKT-E2F-1-RB axis	Breast cancer, Kaposi’s sarcoma etc.
Resisting cell death	Artemisinin and related-derivatives	Malaria	Ferroptosis, autophagy, oncosis, anoikis	Breast, colorectal, lung cancer etc.
Resisting cell death	Chloroquine and related-derivatives	Malaria, rheumatoid arthritis	Autophagy, PPT1	Pancreatic, breast cancer, chondrosarcoma etc.
Enabling replicative immortality	Curcumin	Dermatological diseases	hTERT, Wnt/β-catenin, Hippo/YAP	Breast, prostate cancer, multiple myeloma etc.
Enabling replicative immortality	Genistein	Menopause, osteoporosis, obesity	hTERT, Wnt/β-catenin	Colorectal, bladder, breast cancer etc.
Inducing angiogenesis	Thalidomide	Sedative, antiemetic	Various proangiogenic factors, VEGF receptor, NF-κB	Prostate, ovarian, colorectal cancer etc.
Inducing angiogenesis	Itraconazole	Antifungal agent	mTOR-cholesterol trafficking, VDAC1, PDGF-Akt–mTOR axis	Prostate, lung cancer etc.
Activating invasion and metastasis	Berberine	Bacterial diarrhea	Ephrin-B2, MMP-2/MMP-9, EMT, miR-101, VEGF	Gastric, colorectal, lung cancer etc.
Activating invasion and metastasis	Niclosamide	Antihelminthic drug	Wnt/β-catenin, STAT3, NF-κB	Colorectal, prostate cancer etc.
Genome instability and mutation	Triamterene	Diuretic	Nucleotide excision repair, thymidylate synthase	Acute myelocytic leukemia etc.
Genome instability and mutation	Mebendazole and related-derivatives	Intestinal helminthiasis	Chk2, Nbs1, PARP-1, DHODH	Medulloblastoma, glioma, astrocytoma etc.
Tumor-promoting inflammation	Aspirin	Pain, fever	COX-1/2, ANXA1-NF–κB axis, CDX2, COMMD1–RelA axis	Gastrointestinal, esophageal cancer etc.
Tumor-promoting inflammation	Thiocolchicoside	Rheumatologic, orthopedic disorders	NF-κB-related pathways, COX-2	None
Reprogramming energy metabolism	Metformin	Obese type 2 diabetes	AMPK, PI3K-mTOR pathways, BACH1	Prostate, breast, colorectal cancer etc.
Reprogramming energy metabolism	Disulfiram	Alcohol-aversion drug	ALDH, NAD^+^-dependent proteins	Prostate, breast cancer, melanoma etc.
Evading immune destruction	Rotavirus vaccines, Live 17D	Infectious disease	NF-κΒ, Type I interferon pathways, CTLs, Tregs	None

This table lists existing non-oncology agents for cancer therapy in this review, including targeting cancer hallmarks, original application, reported targets or pathways and related clinical trials in cancer treatment (https://clinicaltrials.gov/).

However, there are some key points that deserve attention. Firstly, cancer is governed by multiple, complex cellular pathways, that could ultimately inhibit the effort of a specific targeted therapy. It is not trivial to replace inoperative targeted drugs with suitable multi-targeted non-oncology drugs, as evidenced by the few regimes currently used to treat cancer patients. So where do we go next? Drug combination therapies, an alternative strategy, should be considered which might have a higher success rate in clinical application. Drug combination therapies usually target multiple mechanisms, including downstream off-target, parallel pathways or compensatory signaling that contribute to tumorigenesis. Next, current cancer treatments mainly focus on directly killing cancer cells in both de novo drug discovery and drug repurposing. This direction seems rationale because increasing programmed cell death, like lethal-autophagy, ferroptosis, pyroptosis etc., are comprehensively studied and can provide alternative strategies for apoptosis-resistant cancer. Nevertheless, residual cancer cells (known as minimal residual disease), often remain after treatment, and are warning that “what does not kill me, makes me stronger”. Thus, using non-oncology drugs that have the potential to target the multiple hallmarks of cancer and the related cancer biology, instead of directly killing the cancer cells per ce heralds them as a key complement to the new paradigm of personalized/precision medicine in the coming future. Meanwhile, the ever-increasing availability of biomedical data from public databases coupled with continued advances in techniques and analytical methods forms a sound basis for the accurate identification of repurposing candidates. In this respect, the PRISM public resource repurposing dataset, an unbiased multi-platform approach recently generated by Corsello et al., will prove a starting point for many future orientation studies.^510,511^

In summary, drug repurposing that opened a window for drug discovery is a trend for including, but not limited to, cancer therapy, regardless of many failures. As Nobel laureate in physiology and medicine, James Black, once said: “the most fruitful basis for the discovery of a new drug is to start with an old drug”.^522^ We totally concur with this concept.
